# Human Metapneumovirus Glycoprotein G Inhibits Innate Immune Responses

**DOI:** 10.1371/journal.ppat.1000077

**Published:** 2008-05-30

**Authors:** Xiaoyong Bao, Tianshuang Liu, Yichu Shan, Kui Li, Roberto P. Garofalo, Antonella Casola

**Affiliations:** 1 Department of Pediatrics, University of Texas Medical Branch, Galveston, Texas, United States of America; 2 Department of Microbiology and Immunology, University of Texas Medical Branch, Galveston, Texas, United States of America; 3 Sealy Center for Vaccine Development, University of Texas Medical Branch, Galveston, Texas, United States of America; University of North Carolina, United States of America

## Abstract

Human metapneumovirus (hMPV) is a leading cause of acute respiratory tract infection in infants, as well as in the elderly and immunocompromised patients. No effective treatment or vaccine for hMPV is currently available. A recombinant hMPV lacking the G protein (rhMPV-ΔG) was recently developed as a potential vaccine candidate and shown to be attenuated in the respiratory tract of a rodent model of infection. The mechanism of its attenuation, as well as the role of G protein in modulation of hMPV-induced cellular responses *in vitro*, as well as *in vivo*, is currently unknown. In this study, we found that rhMPV-ΔG-infected airway epithelial cells produced higher levels of chemokines and type I interferon (IFN) compared to cells infected with rhMPV-WT. Infection of airway epithelial cells with rhMPV-ΔG enhanced activation of transcription factors belonging to the nuclear factor (NF)-κB and interferon regulatory factor (IRF) families, as revealed by increased nuclear translocation and/or phosphorylation of these transcription factors. Compared to rhMPV-WT, rhMPV-ΔG also increased IRF- and NF-κB-dependent gene transcription, which was reversely inhibited by G protein expression. Since RNA helicases have been shown to play a fundamental role in initiating viral-induced cellular signaling, we investigated whether retinoic induced gene (RIG)-I was the target of G protein inhibitory activity. We found that indeed G protein associated with RIG-I and inhibited RIG-I-dependent gene transcription, identifying an important mechanism by which hMPV affects innate immune responses. This is the first study investigating the role of hMPV G protein in cellular signaling and identifies G as an important virulence factor, as it inhibits the production of important immune and antiviral mediators by targeting RIG-I, a major intracellular viral RNA sensor.

## Author Summary

Human metapneumovirus (hMPV), a member of the *Paramyxoviridae* family, is an important cause of respiratory morbidity throughout life. The contribution of viral-specific proteins to the pathogenesis of hMPV infection and immune evasion is largely unknown. Previous work has suggested that the glycoprotein G of hMPV is not necessary for the process of viral fusion and attachment to host cells, and a recombinant hMPV lacking the G protein (rhMPV-ΔG) shows an attenuated phenotype in the respiratory tract of animal models of infection. Airway epithelial cells, a major component of the innate immune system, are a primary target of hMPV infection. In this study, we show that hMPV G protein functions as a major inhibitory factor of the host antiviral response by blocking production of inducible chemokines and IFN-α/β. A major finding of this work is the demonstration that hMPV G protein interacts with RIG-I, a cytoplasmic viral sensor. As result, hMPV G protein inhibits RIG-I-dependent signaling pathways, including activation of NF-κB and IRF-3, two transcription factors necessary for the synthesis of inflammatory and antiviral cytokines. Understanding the function of hMPV proteins is critical for the future design of effective antiviral therapies and rationale design of vaccine candidates.

## Introduction

Human metapneumovirus (hMPV) is a leading cause of both upper and lower respiratory tract infections in infants, elderly and immunocompromised patients worldwide [1]. It is an enveloped, nonsegmented, negative-strand RNA virus, belonging to the *Paramyxoviridae* family, expressing three putative viral membrane proteins, the fusion protein F, the attachment glycoprotein G and the small hydrophobic protein SH [2]. The role of G protein in viral replication was recently investigated both *in vitro* and *in vivo*. Recombinant hMPV (rhMPV) in which the G protein was deleted (rhMPV-ΔG) exhibited reduced replication in the upper and lower respiratory tract of Syrian hamsters and African green monkeys [3],[4]. The mechanism(s) underlying rhMPV-ΔG attenuation, as well as the role of the hMPV G protein in modulating host cell responses are largely unknown.

Sequence analysis of hMPV G protein suggests that it is a type II mucin-like glycosylated protein [2]. The membrane anchor of G protein is proximal to the N termini and its C termini is oriented externally. Although the postulated function of G protein is for attachment, it is not the only attachment protein since hMPV F protein alone is sufficient to mediate attachment and fusion in absence of other surface proteins [3],^5^. Although the reduced attachment ability of G deleted mutant might be the reason for the observed attenuation of rhMPV-ΔG *in vivo*, it is also possible that hMPV G protein may have anti-viral function by suppressing the secretion of pro-inflammatory and/or antiviral molecules upon infection, similar to what has been recently shown for the respiratory syncytial virus (RSV) G protein. RSV lacking G protein induces more cytokines in viral-infected monocytes and human lung epithelial cells, compared to WT RSV [6],[7]. Increased CC and CXC chemokine expression by G protein deletion was also observed in a BALB/c mouse model of RSV infection [8]. The mechanism for RSV G protein anti-inflammatory activity is not clear.

In this study we investigated whether G protein could modulate host innate immune responses following infection with hMPV. Our results show that G protein indeed plays a role in regulating the signaling pathways leading to the production of pro-inflammatory molecules and type I IFN by affecting activation of NF-κB and IRF, two key transcription factors involved in IFN, cytokine and chemokine gene expression.

Two RNA helicases, retinoic inducible gene (RIG)-I and melanoma differentiation associated gene (MDA)-5 have been identified to be essential for IFN induction by several viruses including NDV, Sendai, HCV and RSV [9]–[11]. We have recently shown that RIG-I plays a major role in hMPV-induced cellular signaling [12]. Both RIG-I and MDA-5 share a helicase domain, that is required for their interaction with viral RNA [9],[13], and a CARD domain, that mediates their interaction with the adaptor molecule IPS-1/MAVS/VISA/Cardif, leading to subsequent activation of downstream signaling molecules such as IRFs, NF-κB and AP-1 [14],[15]. In this study, we found that hMPV G protein interacts with RIG-I and blocks RIG-I-, but not MDA-5- or MAVS-dependent gene transcription, identifying G as an important virulence factor, responsible for inhibiting innate immune responses to hMPV infection.

## Results

### Generation of recombinant hMPV

**Figure 1:**
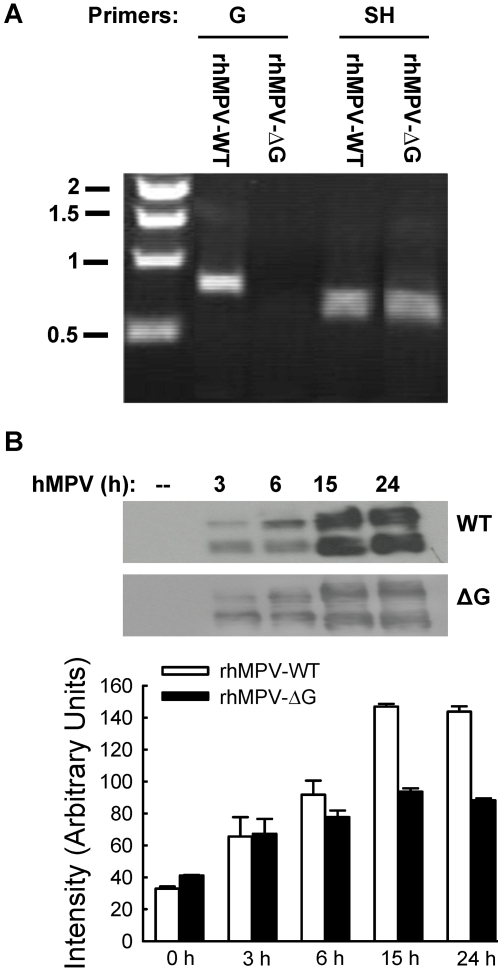
Characterization of recombinant viruses. (A) Verification of G protein deletion. Viral RNA extracted from purified viruses was subjected to RT-PCR using paired primers for G or SH genes. PCR products were then analyzed on a 1% agarose gel. Numbers on the left side represent molecular weight marker size expressed in kilobase (Kb). (B) F protein expression analysis in infected cells. A549 cells were infected with rhMPV-WT or rhMPV-ΔG, MOI of 2, and harvested to prepare total cell lysates at the indicated times. Equal amounts of protein were subjected to SDS-PAGE, followed by Western blot using a monoclonal antibody against hMPV F protein. The results are the representative of three independent experiments. Densitometric analysis of band intensity was performed using the histogram function of Adobe Photoshop.

To investigate the role of hMPV glycoprotein G in hMPV-induced cellular responses, we generated wild-type (WT) recombinant hMPV, as well as hMPV lacking G protein (ΔG), using a reverse genetic system approach [3],[16]. To verify whether G was properly deleted, viral RNA was prepared and subsequently subjected to reverse transcriptase polymerase chain reaction (RT-PCR) using paired primers for G or SH gene amplification, as described in Methods. As expected, there was no band corresponding to amplified G in rhMPV-ΔG, while SH gene, used as positive control, was detected from both rhMPV-WT and rhMPV-ΔG (Fig. 1A).

We next determined whether viral replication of the recombinant viruses was similar to the parental Canadian hMPV83 isolate. To do so, LLC-MK2 cells were infected with hMPV, naïve or recombinant, at MOI of 0.1. At 5 days p.i, the naïve hMPV and rhMPV-WT were harvested. We found that the titers of naïve hMPV and rhMPV-WT were essentially the same, while viral titers of rhMPV-ΔG were slightly lower, between two and three fold less than naïve hMPV and rhMPV-WT at all time points tested. A similar difference was noted when viruses were grown in Vero cells. To determine whether the initial replication phase of the WT and ΔG mutant viruses in airway epithelial cells, the target cell of hMPV infection, was similar, A549 cells were infected with rhMPV-WT or -ΔG at MOI of 2 and harvested at different time post-infection (p.i.) to measure viral titers and determine viral antigen expression. Immunofluorescence analysis using anti-hMPV polyclonal antibody showed that the percentage of cells infected with rhMPV-WT or -ΔG at 15 and 24 h p.i. was similar, around 80–85% (data not shown). However, there was significant less F protein expression in rhMPV-ΔG-infected cells, compared to rhMPV-WT (Fig. 1B), and viral titers at 6, 15 and 24 h p.i. were lower (one third to half log less) in A549 cells infected with rhMPV-ΔG compared to WT virus, indicating a role of G protein in hMPV replication in airway epithelial cells.

### G protein affects hMPV-induced type I interferon and pro-inflammatory cytokine and chemokine secretion *in vitro*

**Figure 2:**
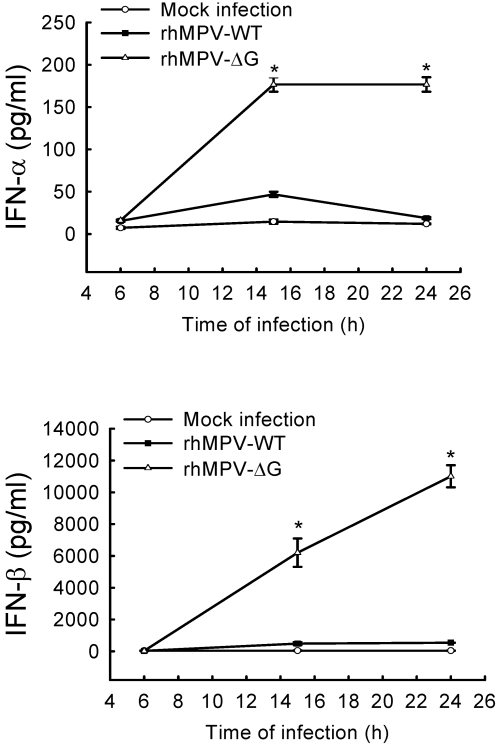
Effect of G protein deletion on type I IFN secretion. A549 cells were infected with rhMPV-WT or rhMPV-ΔG, at MOI of 2, and harvested at 6, 15 and 24 h p.i. to measure secretion of IFN-α and IFN-β in cell supernatants by ELISA. Data shown are representative of three independent experiments. *, *P*<0.05 relative to rhMPV-WT.

It was recently shown that rhMPV-ΔG is highly attenuated in the lower and upper respiratory tract of animal models of infection, compared to rhMPV-WT [3],[4]. Type I interferons play a major role in limiting viral replication [reviewed by [17]]. To determine whether the restriction of rhMPV-ΔG replication *in vivo* could be due to increased IFN-α/β production, airway epithelial cells were infected with either rhMPV-ΔG or rhMPV-WT and cell supernatants were harvested at various time p.i to measure both IFN-α and β by ELISA. As show in Fig. 2, infection of A549 cells with rhMPV-ΔG resulted in a 4-fold and 7-fold increase in IFN-α secretion at 15 h and 24 h p.i respectively, compared to rhMPV-WT. Similarly, IFN-β secretion was 13-fold and 20-fold higher in cells infected with rhMPV-ΔG at 15 h and 24 h p.i. compared to cells infected with rhMPV-WT.

**Figure 3:**
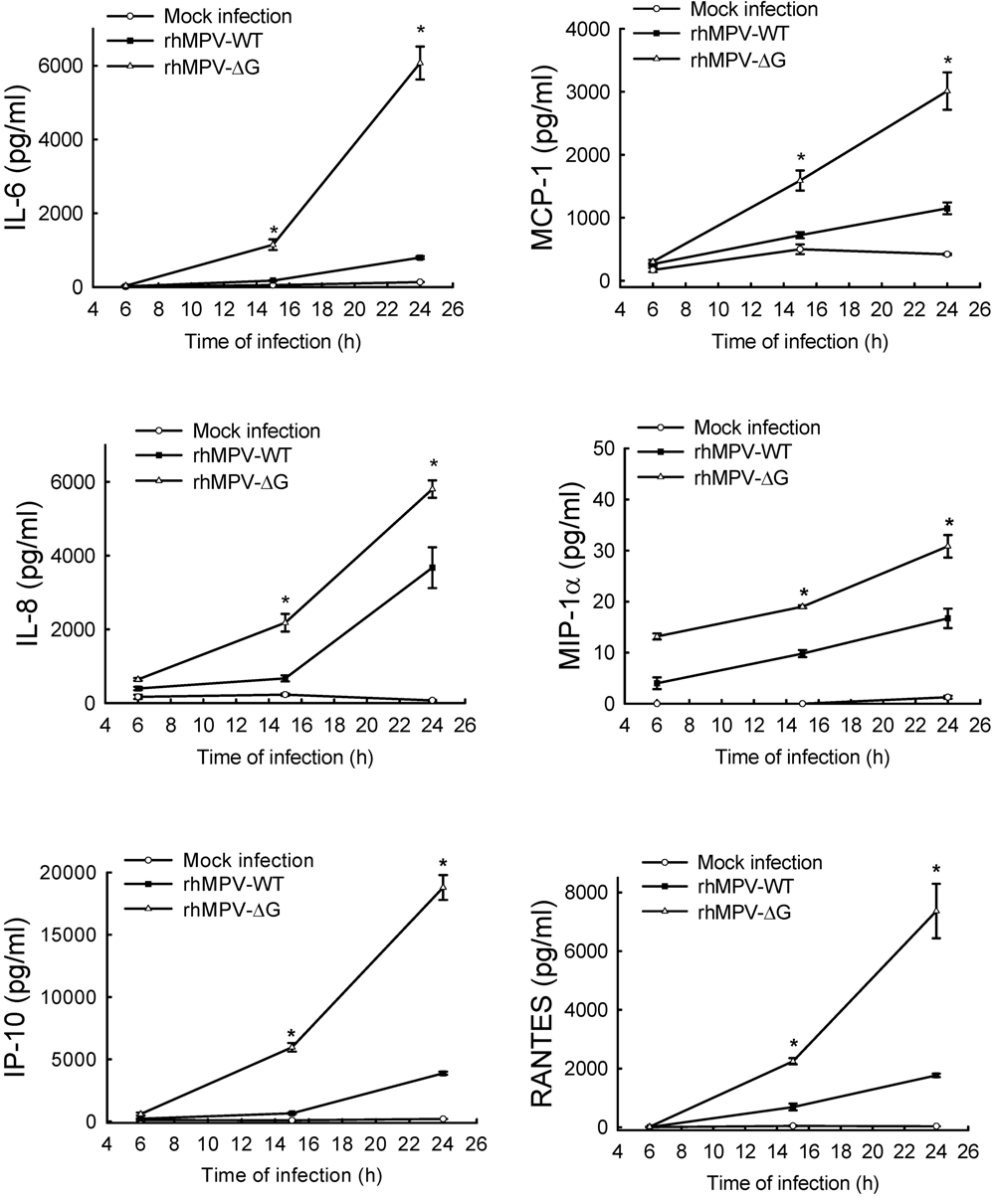
Effect of G protein deletion on cytokine and chemokine secretion. A549 cells were infected with rhMPV-WT or rhMPV-ΔG, at MOI of 2, and harvested at 6, 15 and 24 h p.i. to measure secretion of cytokines, CXC chemokines and CC chemokines in cell supernatants by Bio-Plex. Data shown are representative of three independent experiments. *, *P*<0.05 relative to rhMPV-WT.

To determine whether G protein deletion had a broader effect on hMPV-induced secretion of pro-inflammatory and immunoregulatory molecules, we compared the secretion pattern of chemokines and cytokines in A549 cells infected with either rhMPV-WT or rhMPV-ΔG, using a combination of ELISA and Bio-Plex assays (Fig. 3). rhMPV-ΔG induced significantly higher amounts of the cytokine IL-6, the CXC chemokines IL-8 and IP-10, and CC chemokines MCP-1, MIP-1α and RANTES at both 15 and 24 h p.i, compared to hMPV-WT. A significant difference in IL-8 and MIP-1α induction between rhMPV-WT- and rhMPV- ΔG-infected cells was noted as early as 6 h p.i.

### Modulation of IRF activation by hMPV G protein

Transcription factors of the interferon regulatory factor (IRF) family have been shown to play an essential role in viral-induced expression of type I IFN genes (reviewed in [18]). They also regulate the induction of several other genes involved in the immune/inflammatory response to viral infections, including chemokines, such as RANTES and IP-10, and cytokines, such as IL-15 [Reviewed in [18]]. Among the different members of the IRF family, IRF-1, -3, -5 and -7 have been identified as direct transducers of viral-induced signaling, with IRF-3 being necessary for IFN-β and RANTES gene expression in response to paramyxovirus infections [19].

**Figure 4:**
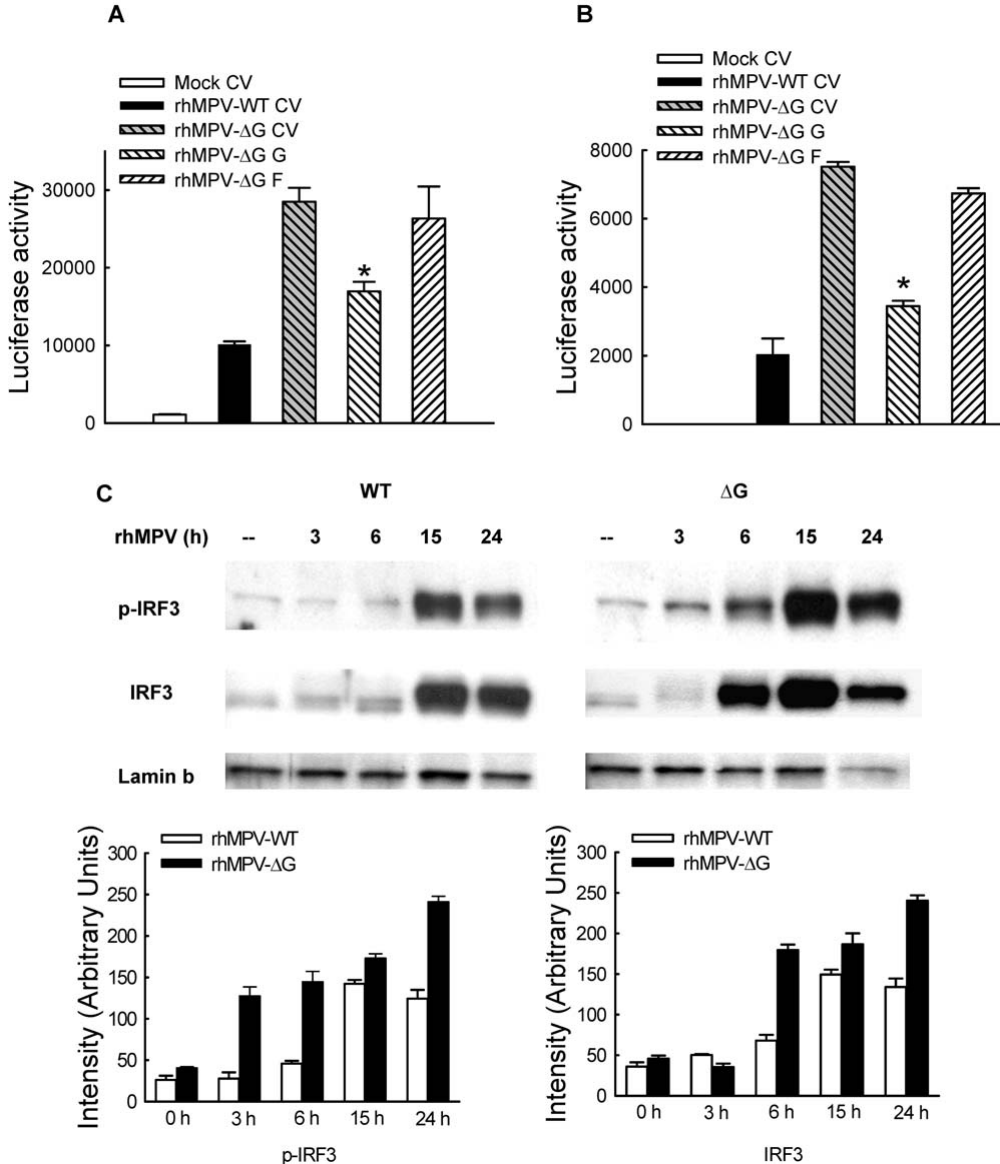
hMPV G protein modulates viral-induced IRF-3 activation. A549 cells were cotransfected with a luciferase reporter plasmid containing either the human IFN-β promoter (A) or multimers of the RANTES ISRE site (B), and the expression plasmid containing hMPV G or F protein or the control vector (CV), and infected with rhMPV-WT or -ΔG, at MOI of 2. Cells were harvested at 15 h p.i. to measure luciferase activity. Uninfected plates served as controls. For each plate luciferase was normalized to the β-galactosidase reporter activity. Data are representative of two independent experiments and are expressed as mean±standard error of normalized luciferase activity. *, *P*<0.05, relative to rhMPV-ΔG-infected-CV transfected A549 cells. (C) A549 cells were infected with rhMPV-WT or rhMPV-ΔG, at MOI of 2, for various lengths of time and harvested to prepare nuclear extracts. Equal amounts of protein from uninfected and infected cells were analyzed by Western blot using either an anti-Ser396 phospho-IRF-3 (pIRF-3) or regular anti-IRF-3 antibody. Membranes were stripped and reprobed for lamin b, as control for equal loading of the samples. Densitometric analysis of IRF band intensity, performed using the histogram function of Adobe Photoshop, is shown after normalization to lamin b.

To investigate the role of G protein in hMPV-induced type I interferon expression and IRF protein activation, we initially determined the effect of G protein deletion on IFN-β gene transcription using transient transfection assays. A549 cells were transfected with a reporter plasmid containing the luciferase gene under control of the IFN-β promoter (IFN-β-LUC) [11], and either mock infected or infected with rhMPV-WT or -ΔG. Cells were harvested at 15 h p.i. to measure luciferase activity. As shown in Fig. 4A, luciferase activity was significantly higher (3 fold) in A549 cells infected for 15 h with rhMPV-ΔG, compared to rhMPV-WT. Enhanced activation of the IFN-β promoter in cells infected with rhMPV-ΔG was also observed at 24 h p.i. (data not shown). To confirm the inhibitory role of G in the induction of IFN-β, we infected A549 cells with rhMPV-ΔG in presence of plasmid expressing either G or F protein. As shown in Fig. 4A, expression of hMPV G but not F protein reversibly inhibited the enhanced IFN-β gene transcription in response to rhMPV-ΔG infection. Similarly, G protein expression significantly decreased IFN-β gene transcription induced by RSV, another paramyxovirus (Fig.S1).

To determine whether G protein deletion specifically affected hMPV-induced IRF-dependent gene transcription, A549 cells were transiently transfected with a construct containing multiple copies of the RANTES ISRE site linked to the luciferase reporter gene [20] and infected with rhMPV-WT or rhMPV-ΔG. Infection of rhMPV-ΔG resulted in significantly higher luciferase activity, compared to rhMPV-WT, which was significantly inhibited by G but not F protein expression (Fig. 4B).

IRF-3 activation is necessary for IFN-β and RANTES gene expression in response to paramyxovirus infections [21],[22]. IRF-3 is constitutively expressed and in unstimulated cells appears in two forms, when resolved by SDS-PAGE, defined as forms I and II [23]. Following viral infections, IRF-3 undergoes a shift in molecular weight and migrates as forms III and IV, due to the virus-induced C-terminal phosphorylation [23], which occurs on specific serine residues and is necessary for IRF nuclear translocation, dimerization, binding to DNA and activation of transcription (Reviewed in [24]). To determine whether changes in abundance of IRF-dependent gene expression, as well as the increased IRF-driven gene transcription observed in response to rhMPV- ΔG infection, resulted from enhanced IRF-3 protein activation, we investigated IRF serine phosphorylation and nuclear translocation by Western blot analysis, using nuclear extracts from control and rhMPV-infected A549 cells (Fig. 4C). IRF-3 phosphorylation occurred earlier and was significantly higher in rhMPV-ΔG infected cells at all time points of infection, compared to rhMPV-WT, which induced IRF-3 phosphorylation only at 15 and 24 h p.i. (Fig. 4C). The enhanced IRF-3 phosphorylation was paralleled by a significant increase in nuclear translocation, also observed at all time points of rhMPV-ΔG infection, compared to rhMPV-WT (Fig. 4C). Enhanced activation was also demonstrated by the shift in molecular weight and slower gel migration of IRF-3 in cells infected with rhMPV-ΔG at 3, 6, 15 and 24 h p.i., compared to rhMPV-WT, in which IRF-3 nuclear translocation and molecular shift occurred only at 15 and 24 h p.i.

### G protein modulates hMPV-induced NF-κB activation

NF-κB is a superfamily of ubiquitous transcription factors composed of NF-kB1 or p50, NF-kB2 or p52, Rel A or p65, RelB and c-Rel proteins, which can form homo- and hetero-dimers and produce complexes with various transcriptional activities [25]. Their activation is controlled by accessory inhibitory proteins called IκBs [26]. NF-κB inducing stimuli cause IκB phosphorylation, through activation of the multicomponent IκB kinase (IKK) complex [25], with subsequent IκB proteolytic degradation [27], event that allows NF-κB to enter the nucleus and activate target gene transcription. A number of paramyxovirus-inducible inflammatory and immunoregulatory genes require NF-κB for their transcription, as we have shown *in vitro* for IL-8 [28], RANTES [22], as well as other chemokines, cytokine, secreted proteins and signaling molecules [29].

**Figure 5:**
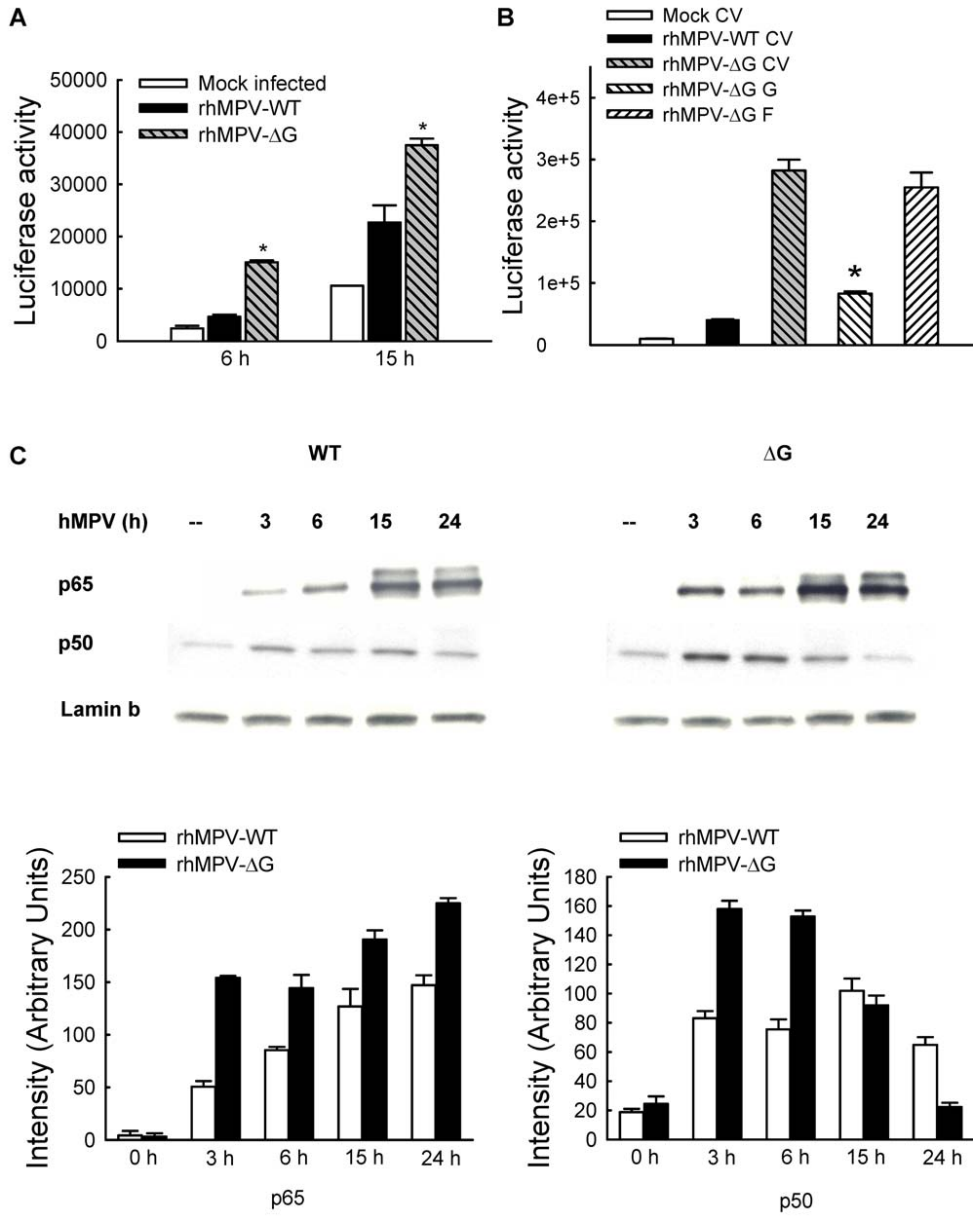
hMPV G protein modulates viral-induced NF-κB activation. (A) A549 cells were transfected with a luciferase reporter plasmid containing the human IL-8 promoter and infected with rhMPV-WT or -ΔG, at MOI of 2. Cells were harvested at 15 h p.i. to measure luciferase activity. Uninfected plates served as controls. For each plate luciferase was normalized to the β-galactosidase reporter activity. Data are representative of two independent experiments and are expressed as mean±standard deviation of normalized luciferase activity. *, *P*<0.05 relative to rhMPV-WT. (B) A549 cells were transfected with a luciferase reporter plasmid containing multimers of the IL-8 NF-κB site together with G or F protein expression plasmid or thes empty vector and infected with rhMPV-WT or -ΔG, at MOI of 2. Cells were harvested at 15 h p.i. to measure luciferase activity. Uninfected plates served as controls. For each plate luciferase was normalized to the β-galactosidase reporter activity. Data are representative of two independent experiments and are expressed as mean±standard deviation of normalized luciferase activity. *, P<0.05 relative to rhMPV-ΔG-infected-CV transfected A549 cells. (C) A549 cells were infected with rhMPV-WT or rhMPV-ΔG, at MOI of 2, for various lengths of time and harvested to prepare nuclear extracts. Equal amounts of protein from uninfected and infected cells were analyzed by Western blot using either an anti-p50 or anti-p65 antibody. Membranes were stripped and reprobed for lamin b, as control for equal loading of the samples. Densitometric analysis of NF-κB band intensity, performed using the histogram function of Adobe Photoshop, is shown after normalization to lamin b.

To determine whether the observed increased in IL-8 production in response to rhMPV-ΔG infection was due to enhanced IL-8 gene transcription, A549 cells were transfected with a construct containing the human IL-8 promoter linked to the luciferase reporter gene [30] and infected with either rhMPV-WT or rhMPV-ΔG. Consistent with IL-8 secretion, rhMPV-ΔG-induced IL-8 promoter activation occurred earlier and was significantly higher (∼3 and 1.8 fold increase at 6 and 15 h p.i.) than rhMPV-WT (Fig. 5A).

To investigate the role of NF-κB in rhMPV-ΔG-induced enhanced IL-8 gene transcription, 293 cells were transiently transfected with a construct containing multiple copies of the IL-8 NF-κB site linked to the luciferase reporter gene [30] and infected with either rhMPV-WT or -ΔG. Compared to rhMPV-WT, infection with rhMPV-ΔG resulted in significant higher NF-κB-driven gene transcription at 6 and 15 h p.i., with the most significant difference occurring at 15 h p.i., which was inhibited by expression of G but not F protein (Fig. 5B).

We have previously show that p65 and p50 are the two major NF-κB family members induced by paramyxovirus infection of airway epithelial cells [28], with p65 being necessary for a variety of viral-induced chemokines and cytokine gene expression [29]. To determine whether G protein plays a role in modulating hMPV-induced NF-κB activation, we investigated p65 and p50 nuclear translocation in A549 cells infected with either rhMPV-WT or -ΔG. Both viruses induced significant p65 and p50 nuclear translocation as early as 3h p.i., however the nuclear abundance of both NF-κB subunits was significantly higher in rhMPV-ΔG infected cells, compared to rhMPV-WT (Fig. 5C).

In conclusion, G protein deletion enhanced hMPV-induced expression of pro-inflammatory and immunoregulatory mediators, likely through enhanced activation of transcription factors belonging to the NF-κB and IRF families.

### hMPV G inhibits RIG-I-mediated activation of the IFN-β promoter

**Figure 6:**
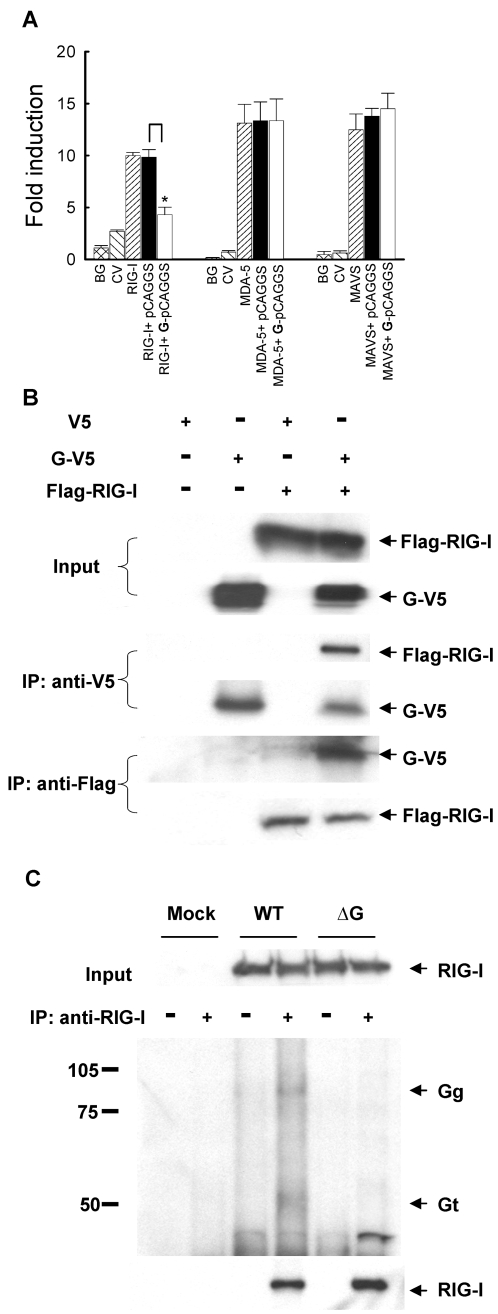
Inhibition of RIG-I-mediated signaling by G protein. (A) A549 cells were transfected with a luciferase reporter plasmid containing the human IFN-β promoter, plasmids encoding either RIG-I, MDA-5 or MAVS or their control vectors, and a plasmid expressing hMPV G or the empty vector (as indicated at the bottom of each column). Cells were harvested 30 h post-transfection to measure luciferase activity. For each plate luciferase was normalized to the â-galactosidase reporter activity. Data are representative of two independent experiments and are expressed as mean±SD of normalized luciferase activity. *, P<0.05 relative to pCAGGS+RIG-I group. BG: background; CV: control vector. (B) 293 cells were transfected with plasmids encoding Flag-tagged RIG-I and V5-tagged G or their control vectors. Total cell lysates were immunoprecipitated with anti-V5 antibody followed by Western blot using anti-Flag antibody to detect RIG-I. Reverse immunoprecipitation was also done, where RIG-I was immunoprecipitated using anti-Flag antibody and G protein was then detected using anti-V5 antibody. Membranes were stripped and reprobed to check for proper expression of G and RIG-I. (C) A549 cells were mock infected or infected with rhMPV-WT or -ΔG, MOI of 2, and harvested at 24 h p.i. to prepare total cell lysates. Samples were subjected to immunoprecipitation using anti-RIG-I antibody or control isotype. The immunoprecipitated complexes were then subjected to SDS-PAGE followed by Western blot using anti-hMPV antibody. Membrane was then stripped and reprobed with anti-RIG-I antibody to determine levels of immunoprecipitated RIG-I. Numbers on the left side represent molecular weight marker size expressed in kilodalton. Arrows indicate bands corresponding to hMPV G protein.

The RIG-I/MDA-5/MAVS pathway plays an essential role for initiating cellular signals leading to the activation of transcription factors and subsequent induction of type I IFN in the course of viral infections [13],[31],[32]. In recent investigations, we found this pathway is necessary for hMPV-induced gene expression in airway epithelial cells [12]. Therefore, we investigated whether the G protein inhibitory effect on NF-κB and IRF activation and subsequent induction of pro-inflammatory and anti-viral molecules occurred via inhibiting RNA helicase-initiated cellular signaling. To do so, A549 cells were transfected with Flag-tagged RIG-I, MDA-5 or MAVS expression plasmids with a plasmid containing the luciferase reporter gene under the control of the IFN-β promoter (IFN-β-Luc). Individual expression of all three signaling molecules significantly induced IFN-β promoter activation, in the absence of viral infection (Fig. 6A). RIG-I-dependent IFN-β promoter activation was inhibited by G protein expression, while there was no effect on MDA-5- or MAVS-induced luciferase activity (Fig. 6A).

Next, we investigated whether G protein was able to directly interact with RIG-I, disrupting its ability to mediate cellular signaling, as it was recently shown for influenza virus NS1 protein [33]–[35]. 293 cells were transfected with V5-tagged G and Flag-tagged RIG-I expression plasmids. Vectors expressing V5 or Flag only were used as negative controls. After 30 h of transfection, cells were lysed followed by immunoprecipitation using anti-V5 antibody (Fig. 6B). The immunoprecipitated complex was separated on SDS-PAGE and transferred onto PVDF membrane. Western blot using anti-Flag antibody revealed that RIG-I coprecipitated with G protein. Reverse immunoprecipitation, using anti-Flag to precipitate expressed RIG-I and then using anti-V5 antibody for Western blot also confirmed that G was present in the immuno-precipitated complex (Fig. 6B). A similar experiment performed using a V5-tagged F expression plasmid, instead of G, and Flag-tagged RIG-I did not show any interaction between the two proteins (Fig. S2).

To confirm that G interacts with RIG-I in conditions of naïve protein expression, A549 cells were mock infected or infected with rhMPV-WT or rhMPV-ΔG. For each condition, half sample was coimmunoprecipitated using anti-RIG-I antibody, while the other half was exposed to an isotype antibody to rule out nonspecific protein binding during the immunoprecipitation. Two specific bands around 90 and 50 kD were detected in rhMPV-WT-infected samples, but not in the mock infected or rhMPV-ΔG samples, using an anti-hMPV antiserum, likely corresponding to the two major glycosylated forms of G protein (Fig. 6C), as described in [3]. The same size bands were detected by the anti-hMPV antiserum when G was expressed in 293 cells, while the F protein, used as a control, run with a different molecular weight (Fig. S3).

## Discussion

The innate immune response represents a critical component of the host defense against viruses and is coordinated at the cellular level by activation of transcription factors that regulate the expression of inducible gene products with antiviral and/or inflammatory activity. As the immune system evolved to fight viral infections, so viruses developed strategies to evade the host immune responses, mainly by targeting the type I interferon system. HMPV is the second most common cause of epidemic respiratory infections in infants and young children and a significant cause of respiratory tract infections in the elderly and immunocompromised patients. The availability of the reverse genetic system for negative sense RNA viruses has allowed the dissection of viral protein functions in viral replication as well as in cellular signaling. As a recently identified virus, little is known about the role of individual hMPV proteins in modulating host cell responses. In this study, we found that hMPV infection of airway epithelial cells, the primary target of hMPV infection [36],[37], induced the secretion of a variety of cytokines and chemokines, as well as type I interferons, whose expression is coordinated by subsets of transcription factors belonging to the NF-κB and IRF families. Surprisingly, deletion of G protein resulted in enhanced production of chemokines and type I interferon (IFN), as well as increased activation of both families of transcription factors. The enhanced responses to rhMPV-ΔG infection were not due to an increased ability of rhMPV-ΔG to replicate, as the accumulation of F protein in infected airway epithelial cells and viral titers were lower in cells infected with rhMPV-ΔG, compared to rhMPV-WT.

Circumvention of the IFN response occurs through different strategies. Two major categories include either direct suppression of IFN production or interference with IFN signaling, through inhibition of the JAK/STAT pathway. The kinetics of enhanced chemokine and IFN production and transcription factor activation in response to rhMPV-ΔG infection, which occurred at early time points of infection, suggested that G protein regulated an early signaling event triggered in response to hMPV infection of airway epithelial cells.

RIG-I and MDA-5 are two RNA helicases that have been recently identified as fundamental sensors of viral infections [10],[38]. They recognize single and double-stranded RNA and their engagement triggers a signaling cascade leading to NF-κB and IRF activation, through interaction with the mitochondrial antiviral signaling (MAVS) adaptor molecule [39]. Several viral proteins have been shown to be able to disrupt the RIG-I/MDA-5/MAVS signaling pathway by sequestering viral RNA from helicase binding and/or disrupting helicase interaction with downstream signaling molecules or by increasing protein degradation. Influenza A NS1 protein employs the first two mechanisms to block RIG-I-mediated IFN induction [40]. Similarly, V proteins of paramyxoviruses have been shown to bind MDA-5 and inhibit IFN-β production [13] [41]. On the other hand, poliovirus infection induces MDA-5 degradation, also inhibiting IFN induction [42],[43]. HCV 3/4A proteases cleave MAVS at the level of mitochondria insertion, releasing the protein to the cytoplasmic compartment, therefore preventing further transmission of RIG-I-dependent signaling, resulting in the inhibition of host's antiviral responses [39]. In this study, we found that hMPV G protein physically interacts with RIG-I and inhibits RIG-I, but not MDA-5 and MAVS-induced IFN-β transcription, justifying the broad inhibitory effect of G protein on activation of NF-κB and IRF transcription factors and induction of antiviral and pro-inflammatory molecules.

The inhibitory effect of hMPV G protein on cellular signaling could be a common feature of surface glycoproteins of enveloped single strand, negative strand RNA viruses. RSV G protein has been shown to modulate cytokine and chemokine production, as infection with a mutant RSV lacking the full-length G protein or the soluble part of G protein (sG) enhanced production of IL-6 and IL-8 in monocytes [6], as well as IL-8 and RANTES secretion and ICAM expression in airway epithelial cells [7]. RSV-ΔG also caused more lung inflammation, compared to WT, in a mouse model of infection [6]. The G protein of pneumonia virus of mice, a murine relative of RSV, has also been recently identified as an important virulence factor, as viral replication of a recombinant mutant virus lacking G is severely restricted in a BALB/c mouse model of infection [44]. Similarly, the surface glycoproteins of hantaviruses, in particular the ones associated with hemorrhagic pulmonary syndrome (HPS), have been shown to affect IRF-3 activation and IFN production, via interaction with RIG-I and TBK-1, a kinase responsible for viral-induced IRF-3 phosphorylation, as well as to inhibit IFN-mediated cellular responses [45],[46].

hPMV infection is associated with production of inflammatory mediators not only *in vitro* but also *in vivo* [47]. The high attenuation of rhMPV-ΔG replication in the lower respiratory tracts of rodent models of infection suggest that it could be developed as a vaccine candidate [3] [4]. Our findings suggest that careful investigation is needed in an animal model of infection to address whether rhMPV-ΔG can cause enhance lung inflammation and impaired lung function, as recently shown for RSV lacking the G protein [6],[48].

In summary, this study provides us with novel information on the role of hMPV G protein in regulating host cell responses. Further studies are needed to determine the exact mechanism by which G protein inhibits RIG-I activation and to define the domains/amino acid residues mediating RIG-I and G interaction.

## Materials and Methods

### Viral preparation

LLC-MK2 cells (ATCC, Manassas, VA) were maintained in MEM (Invitrogen GIBCO, Carlsbad, CA) supplemented with 10% fetal bovine serum and Penicillin and Streptomycin (100 U/ml). The Canadian isolate hMPV83 and its derived recombinant viruses were propagated in LLC-MK2 cells at 35°C in the absence of serum and in the presence of 1 µg of trypsin/ml (Worthington, Lakewood, NJ), and were sucrose purified, as previously described [47]. Viral titer was determined by immunostaining in LLC-MK2 cells, as previously described [49].

### Cell culture and infection of epithelial cells with hMPV

A549, human alveolar type II-like epithelial cells, and 293, a human embryonic kidney epithelial cell line (both from ATCC, Manassas, VA), were maintained in F12K and MEM medium respectively, containing 10% (v/v) FBS, 10 mM glutamine, 100 IU/ml penicillin and 100 µg/ml streptomycin. Cell monolayers were infected with hMPV at multiplicity of infection (MOI) of 2 in serum-free medium containing antibiotics and 1 µg of trypsin/ml for all experiment, unless otherwise stated. An equivalent amount of sucrose solution was added to uninfected A549 cells, as control. BSR T7/5 cells, baby hamster kidney cells that constitutively expressing the T7 RNA polymerase, were a gift from Dr. Conzelmann, Munich, Germany. They were maintained in Glasgow minimal essential medium (MEM) supplemented with 1% amino acids, 10% fetal bovine serum, 12 mg/L tryptose phosphate broth, 1 mg/ml of Geneticin, and 100 U/ml of Penicillin and Streptomycin.

### Construction antigenome and support protein plasmids for viral recovery

A plasmid encoding hMPV antigenome was constructed as described in Biacchesi *et al.* [16], with some modifications. Briefly, viral RNA was purified from hMPV CAN83-infected LLC-MK2 cells using QIAamp viral RNA kit (Qiagen, Alameda, CA). First strand cDNA was then generated using Superscript III reverse transcriptase (Invitrogen Carlsbad, CA). PCR was carried out using Pfu DNA polymerase (Stratagene, La Jolla, CA) following manufacturer's instruction. The complete hMPV antigenome cloned in pBSKSII vector (a gift from Dr. Buchholz, NIAID, Bethesda) was obtained by sequential ligation of three fragments amplified from the cDNA template. Fragment I was first cloned using forward primer: 5′-ACGCGAAAAAAACGCGTATAAATTAAGTTAC-3′ and reverse primer: 5′- TTTGTCCCGTTCTTGATT*gctAgC*ATTCTTATTCTAACTTg-3′. The cloned fragment which contains the putative N, P, and M genes was first cloned into TOPO cloning vector (Invitrogen, Carlsbad, CA). On the downstream end of this fragment, an *Nhe*I site was created by four nucleotide substitutions in the putative M–F intergenic region as a marker to distinguish between cDNA-derived and biologically derived hMPV. In order to insert a T7 RNA polymerase promoter (T7p) at the 3′ end of the antigenome, fragment I inserted TOPO was used as template to clone the T7p-fragment I using same reverse primer and forward primer: 5′-cgc*gacgtc* TAATACGACTCACTATAGGGACGCGAAAAAAACGCGTATAAATTAAGTTAC-3′. Italicized letters indicate restriction enzyme site. Underlined letters indicate T7 RNA polymerase promoter (T7p) sequence. Construction of fragment II, which contains F, M2, SH and G) and fragment III, containing L and part of the hepatitis delta virus ribozyme, was done as described in Biacchesi *et al.* [16]. Construction of G deleted mutant cDNA was done as described in Biacchesi *et al.* [3].

hMPV cDNA was also used as a template to clone the support genes necessary for the virus recovery, as described in Biacchesi *et al.* [16]. Amplified PCR fragments, encoding N, P and M2-1, were first inserted into TOPO cloning vector (Invitrogen, Carlsbad, CA), and then restricted with AflIII/XhoI. PCR fragments were then subcloned into NCoI/XhoI digested PTM1, a T7 promoter containing vector (a generous gift from Dr. Moss at NIH). For the N protein, site-directed mutagenesis was performed to silently mutate an AflIII site before cloning the gene into PTM1. To clone the polymerase L, full length L cloned in TOPO vector was cut first with BamHI and blunt ended using T4 DNA polymerase (Invitrogen, Carlsbad, CA) following manufacturer's instruction. PTM1 was also cut first with NcoI and then blunted using T4 DNA polymerase as well. Both vectors were then cut with XhoI and the L fragment was cloned into the blunted NcoI and cohesive XhoI site of PTM1. The plasmids containing the full-length hMPV-WT or -ΔG antigenome or individual support genes were sequenced to verify the absence of significant mutations. Primer sequence for cloning the recombinant viruses, as well as the support viral proteins, are available upon request.

### Recovery of recombinant hMPV

Confluent BSR T7/5 cells in six-well dishes were transfected with 5 µg of antigenomic plasmid corresponding to hMPV wild-type (rhMPV-WT), or one lacking G (rhMPV-ΔG) gene, together with 1–2 µg of PTM1 expressing P and N proteins, and 0.5–1 µg of PTM1 expressing M2-1 and L proteins. Lipofectamine 2000 (Invitrogen) was used as transfection reagent, according to manufacturer's instructions. After overnight incubation at 35°C, transfection medium was removed and replaced with Glasgow MEM without trypsin or serum. Trypsin was added on day 3 post-transfection to a final concentration of 1 µg/ml, then cell-medium mixtures were passaged onto fresh LLC-MK2 cells and incubated at 35°C for the next few days. Typical viral CPE were usually observed around day 5–6 post-infection (p.i.). Recombinant virus generation was confirmed by restriction digestion and the sequencing of viral RNA, as described in Biacchesi *et al.* [16]. The recovered viruses were then amplified for two passages in LLC-MK2 cells and saved as stock viral preparations. Viruses with no more than 4–5 passages were used in all experiments.

### Plasmid construction for G expression

G protein was cloned using the plasmid encoding hMPV antigenome as a template. PCR was carried out using Pfu DNA polymerase (Stratagene, La Jolla, CA) following manufacturer's instruction. The primer sequences for V5-tagged G were: forward: 5′- ACGC*gaattc*ATGGAGGTGAAAGTAGAGAA-3′, and reverse: 5′- T*ctcgag*TCACGTAGAATCGAGACCGAGGAGAGGGTTAGGGATAGGCTTACCGTTTTGCATTGTGCTTACAGATGCCTG-3′. Italicized letters indicate restriction enzyme site. Underlined letters indicate V5 sequence. The cloned V5-tagged G was first cloned into the TOPO cloning vector, and then cut by EcoRI and XhoI and subcloned into pCAGGS vector. Sequence integrity was verified by sequencing performed by the protein chemistry laboratory at UTMB.

### Reporter gene assays

Plasmids containing either the IFN-β promoter, as well as plasmids containing multiple copies of the IL-8 NF-κB site or of the RANTES ISRE site, linked to the luciferase reporter gene, have been previously described [11],[20],[22],[30]. Logarithmically growing A549 or 293 cells were transfected in triplicate with the reporter plasmids using FuGene 6 (Roche, Indianapolis, IN), as previously described [22]. The next day cells were infected with recombinant hMPV, either WT or ΔG, at MOI of 2. Uninfected plates served as control. At various times post-infection, cells were lysed to measure independently luciferase and β-galactosidase reporter activity, as previously described [50]. Luciferase was normalized to the internal control β-galactosidase activity. All experiments were performed in duplicate or triplicate. In the experiments, in which the role of overexpressed G in modulating viral-induced response was investigated, a plasmid encoding G or its control vector was cotransfected with these luciferase reporter genes.

### Coimmunoprecipitation

293 cells with 60–70% confluence in 6-well plate were cotransfected with 0.5 µg of pcDNA6 containing Flag-tagged RIG-I and 0.1 µg of either pCAGGS encoding V5-tagged G protein or the empty vector, used as a negative control. Cells were harvested 30 h after transfection and immunoprecipitation was carried out using immunoprecipitation Kit from Roche (Cat # 11719, Indianapolis, IN). In brief, 6×10^6^ cells were lysed using 1.5 ml of lysis buffer. To reduce background caused by non-specific adsorption of irrelevant cellular proteins to protein A/G-agarose, a preclearing step was performed by incubating the sample with 50 µl of the protein A/G-agarose for 3 h at 4°C on a rocking platform. Precleared samples were then exposed to 5 µg of antibody against either V5 (Invitrogen, Carlsbad, CA) or Flag (Sigma, St. Louis, MO) or to an isotype antibody control, for 1 h at 4°C. 50 µl of the protein A/G-agarose were added to the samples and incubated overnight at 4°C. Immunecomplexes were recovered by centrifugation and washed three times using buffers with different ion strength provided by the kit. The immunoprecipitated complexes were eluted from the beads and subjected to SDS-PAGE and Western blot analysis. To investigate the interaction of endogenous RIG-I with G protein during hMPV infection, confluent A549 cells in 6-well plate were mock infected or infected with rhMPV-WT or -ΔG, at MOI of 2 for 24 h. Cells were lysed and immunoprecipitated using anti-RIG-I antibody (Abgent, San Diego, CA, cat.#AP1900a) or isotype control antibody, as described above. The presence of G protein in the complex was then detected using polyclonal anti-hMPV antibody, a gift from Medimmune, Mountain View, CA.

### Western blot

Nuclear extracts of uninfected and infected cells were prepared using hypotonic/nonionic detergent lysis, according to Schaffner protocol [51]. To prevent contamination with cytoplasmic proteins, isolated nuclei were purified by centrifugation through 1.7 M sucrose buffer A for 30 min, at 12,000 rpm, before nuclear protein extraction, as previously described [52]. Total cell lysates of uninfected and infected cells were prepared by adding ice-cold lysis buffer (50 mM Tric-HCl, pH 7.4, 150 mM NaCl, 1 mM EGTA, 0.25% sodium deoxycholate, 1 mM Na_3_VO_4_, 1 mM NaF, 1% Triton X-100 and 1 µg/ml of aprotinin, leupeptin and pepstatin). After incubation on ice for 10 min, the lysates were collected and detergent insoluble materials were removed by centrifugation at 4°C at 14,000 g. After normalizing for protein content, using Bio-Rad, Hercules, CA. Nuclear extracts or total cell lysates were fractionated by SDS-PAGE, and transferred to polyvinylidene difluoride membranes. Membranes were blocked with 5% milk in TBS-Tween and incubated with the proper primary antibodies according to manufacturer's instruction. Primary antibodies used for IRF-3 and phosphorylated IRF-3 detection was from Santa Cruz (Santa Cruz Biotechnology, Inc., Santa Cruz, CA) and Upstate (Upstate, Lake Placid, NY) respectively. Rabbit anti-p65 and p50 were purchase from Cell Signaling (Cell Signaling Technology, Inc, Danvers, MA). Monoclonal anti-hMPV F was a gift from Medimmune, Mountain View, CA. Proper horseradish-coupled secondary antibody was then used and proteins detected by enhanced chemiluminescence assay (Amersham, Piscataway, NJ).

### Bio-Plex and ELISA

IFN-α and -β concentrations were determined by commercial enzyme-linked immunosorbent assays (ELISA), according to the manufacturer's instructions (PBL, Piscataway, NJ). Chemokines and cytokines (IL-1RA, IL-1β, IL-2, IL-4, IL-5, IL-6, IL-7, IL-9, IL-10, IL-12 p70, IL-13, IL-17, G-CSF, GM-CSF, IFN-γ, IP-10, EOTAXIN, MIP-1α, MIP-1β, GCSF, FGFB, PDGF, VEGF and TNF-α) were quantified by Luminex-based Bio-Plex system (Bio-Rad Laboratories, Hercules, CA) according to the manufacturer's instructions. The lower limit of detection for all cytokines measured by Bio-Plex is 3 pg/ml.

### Statistical analysis

Statistical significance was analyzed by using analysis of variance (ANOVA). *P* value of less than 0.05 was considered significant. Results are shown as mean±SEM.

### Supplementary materials and methods

#### Recombinant human RSV preparation

Vero cells (ATCC, Manassas, VA) were maintained in MEM (Invitrogen GIBCO, Carlsbad, CA) supplemented with 5% fetal bovine serum and Penicillin and Streptomycin (100 U/ml). A2 strain derived human recombinant RSV lacking NS1 and NS2 was kindly provided by Dr. Hong (Medimmune, Gaithersburg, MD) and was propagated in Vero cells at 35°C in the presence of 1% fetal bovine serum. The viruses were sucrose purified, as previously described [47].

#### Plasmid construction for F expression

F protein was cloned using the plasmid encoding hMPV antigenome as a template. PCR was carried out using Pfu DNA polymerase (Stratagene, La Jolla, CA) following manufacturer's instruction. The primer sequences for F were: forward: 5′- ACGC*gaattc*ATGTCTTGGAAAGTGGT -3′, and reverse: 5′- T*ctcgag*ACTGTGTGGTA TGAAGC -3′. Italicized letters indicate restriction enzyme site. The cloned F was first cloned into the TOPO cloning vector, and then cut by EcoRI and XhoI and subcloned in frame into pcDNA6 vector containing V5. The V5-tagged F then was cut from pcDNA6 and subcloned into pCAGGS vector. Sequence integrity was verified by sequencing performed by the protein chemistry laboratory at UTMB.

## Supporting Information

Figure S1 G protein inhibits rhRSV-ΔNS1/NS2-induced IFN-β transcription. Logarithmically growing A549 cells were transfected with a luciferase reporter plasmid containing the human IFN-β promoter together with a plasmid expressing hMPV G or the empty vector. After 24 h post transfection, cells were mock infected or infected with recombinant human RSV lacking NS1 and NS2 (rhRSV-ΔNS1/NS2) at MOI of 1. Cells were harvested at 24 h p.i. to measure luciferase activity. For each plate luciferase was normalized to the β-galactosidase reporter activity. Data are expressed as mean±SD of normalized luciferase activity. CV: control vector. *, *P*<0.05 relative to CV+ rhRSV-ΔNS1/NS2.
(0.37 MB TIF)

Figure S2 RIG-I does not interact with hMPV F protein. 293 cells were transfected with plasmids encoding Flag-tagged RIG-I and V5-tagged F or their control vectors. Total cell lysates were immunoprecipitated with anti-Flag antibody followed by Western blot using anti-V5 antibody to detect hMPV F. Membranes were stripped and reprobed to check for proper expression and immunoprecipitation of RIG-I.
(1.54 MB TIF)

Figure S3 hMPV G and F proteins migrate differently on SDS-PAGE. 293 cells were transfected with a plasmid encoding G (lane 2) or F (lane 4) or their control vector (lane 1 and 3). At 30 h post-transfection, cells were harvested using SDS sample buffer. G and F protein expression was then detected using an anti-hMPV polyclonal antibody.
(0.79 MB TIF)
