# Supplementary figures and images for: Human Metapneumovirus Glycoprotein G Inhibits Innate Immune Responses
Source: PLoS Pathog. 2008 May 30;4(5):e1000077. doi: 10.1371/journal.ppat.1000077 (PMC2386556; doi:10.1371/journal.ppat.1000077)

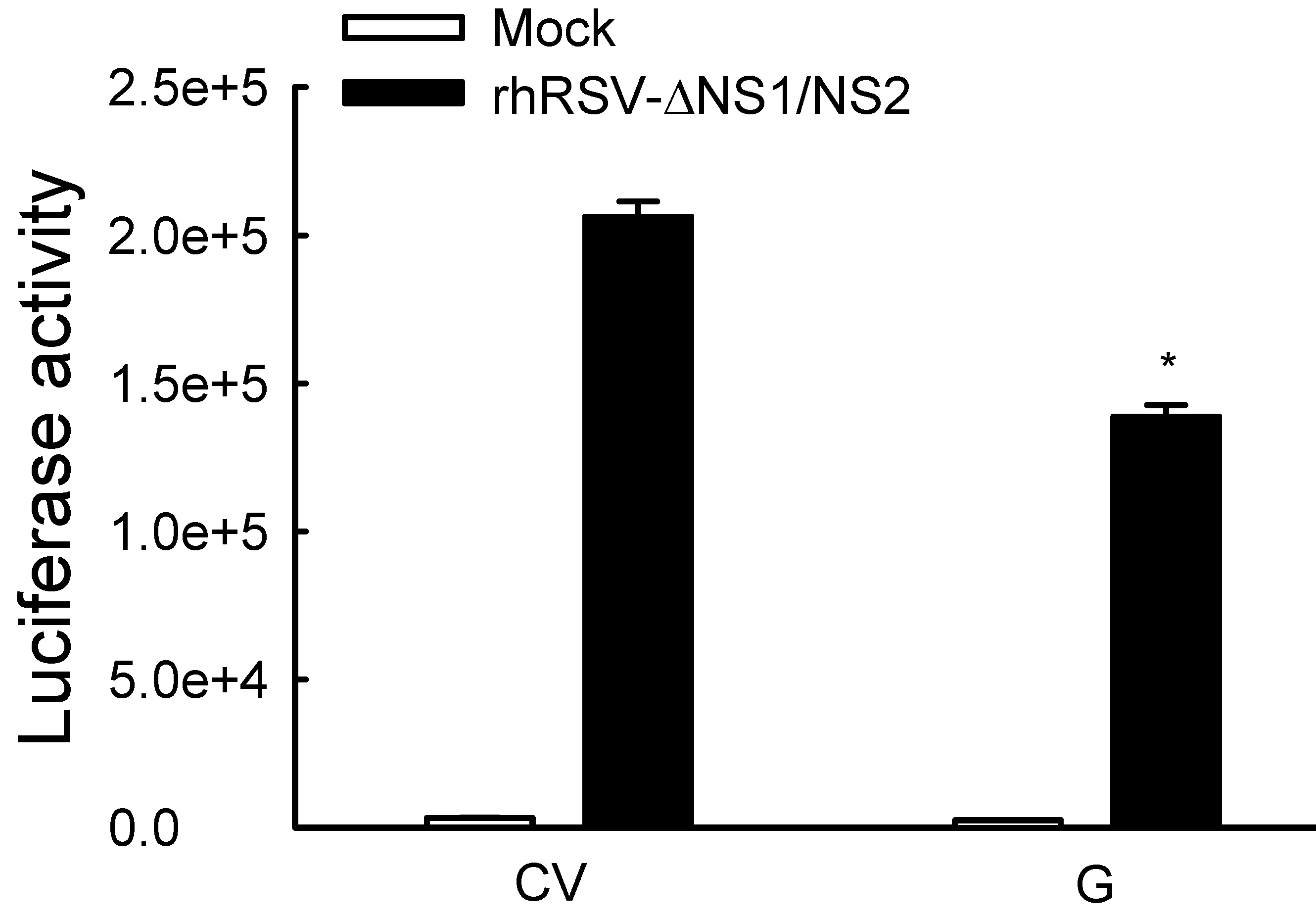

Supplement: Figure S1 — G protein inhibits rhRSV-ΔNS1/NS2-induced IFN-β transcription. Logarithmically growing A549 cells were transfected with a luciferase reporter plasmid containing the human IFN-β promoter together with a plasmid expressing hMPV G or the empty vector. After 24 h post transfection, cells were mock infected or infected with recombinant human RSV lacking NS1 and NS2 (rhRSV-ΔNS1/NS2) at MOI of 1. Cells were harvested at 24 h p.i. to measure luciferase activity. For each plate luciferase was normalized to the β-galactosidase reporter activity. Data are expressed as mean±SD of normalized luciferase activity. CV: control vector. *, P<0.05 relative to CV+ rhRSV-ΔNS1/NS2. (0.37 MB TIF) [file ppat.1000077.s001.tif]

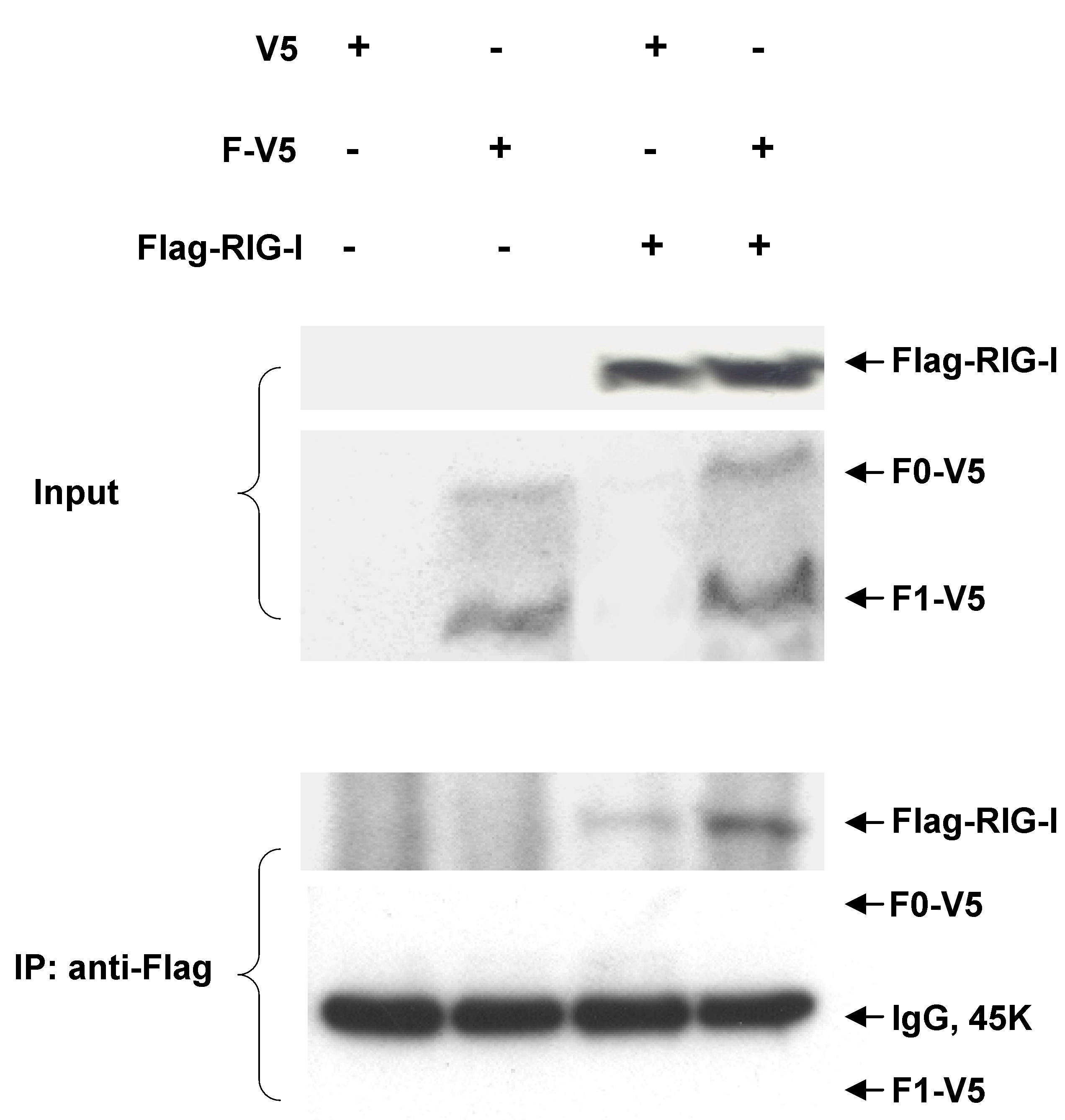

Supplement: Figure S2 — RIG-I does not interact with hMPV F protein. 293 cells were transfected with plasmids encoding Flag-tagged RIG-I and V5-tagged F or their control vectors. Total cell lysates were immunoprecipitated with anti-Flag antibody followed by Western blot using anti-V5 antibody to detect hMPV F. Membranes were stripped and reprobed to check for proper expression and immunoprecipitation of RIG-I. (1.54 MB TIF) [file ppat.1000077.s002.tif]

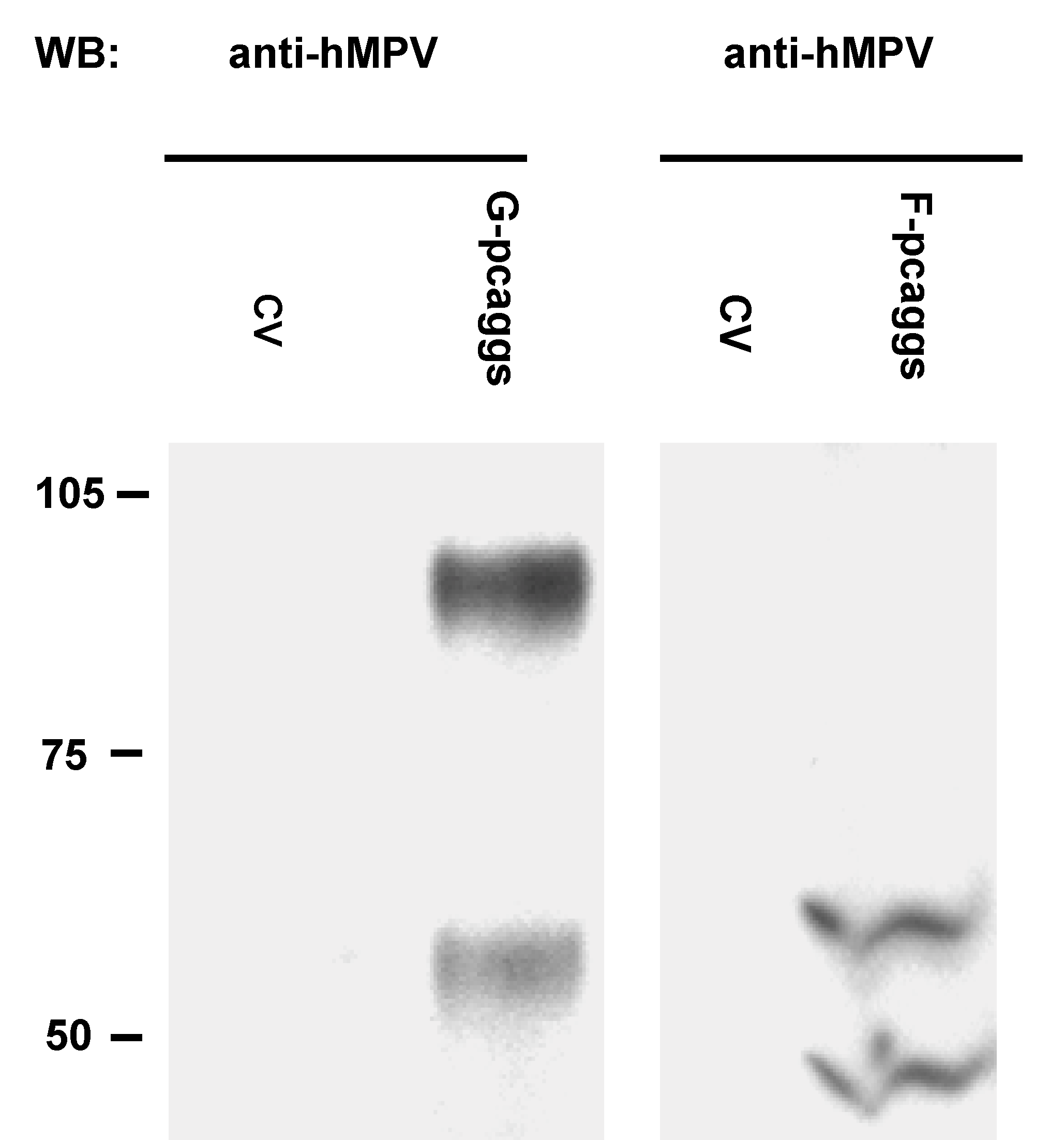

Supplement: Figure S3 — hMPV G and F proteins migrate differently on SDS-PAGE. 293 cells were transfected with a plasmid encoding G (lane 2) or F (lane 4) or their control vector (lane 1 and 3). At 30 h post-transfection, cells were harvested using SDS sample buffer. G and F protein expression was then detected using an anti-hMPV polyclonal antibody. (0.79 MB TIF) [file ppat.1000077.s003.tif]
